# Apatinib enhances chemosensitivity of ABT‐199 in diffuse large B‐cell lymphoma

**DOI:** 10.1002/1878-0261.13309

**Published:** 2022-09-07

**Authors:** Yuanfei Shi, Jing Ye, Huafei Shen, Yi Xu, Rui Wan, Xiujin Ye, Jie Jin, Wanzhuo Xie

**Affiliations:** ^1^ Department of Hematology, the First Affiliated Hospital, College of Medicine Zhejiang University Hangzhou China; ^2^ Sports Medicine Department, Beijing Key Laboratory of Sports Injuries Peking University Third Hospital Beijing China; ^3^ Institute of Sports Medicine of Peking University Beijing China; ^4^ Department of Intensive Care Unit, Taihe Hospital Hubei University of Medicine Shiyan China

**Keywords:** ABT‐199, Apatinib, DLBCL, EDN1, MAPK, VEGFR‐2

## Abstract

To investigate the effect of Apatinib (an inhibitor targeting VEGFR‐2) enhances chemosensitivity of ABT‐199 on diffuse large B‐cell lymphoma (DLBCL). Viability assay and flow cytometric assay for determining apoptosis, cell cycle, mitochondrial membrane potential, reactive oxygen species and immunoblotting were used to explore the combination effect in DLBCL cell lines, DLBCL patient samples, and DLBCL mouse models. RNA sequencing assay helped identify mechanisms of ABT‐199 plus Apatinib. The results show that ABT‐199 combined with Apatinib inhibited cell proliferation, reduced colony‐forming capacity, and induced apoptosis and cell cycle arrest in DLBCL cells. Mechanistically, the combination therapy inhibited tumour cell growth and promoted tumour cell death by regulating EDN1 and MAPK‐related pathways and activating the intrinsic apoptotic pathway. The effect of the combination therapy was also validated in primary DLBCL blasts and xenograft mouse models. Our findings indicate that Apatinib enhances the chemosensitivity of ABT‐199 and might serve as a promising therapeutic strategy for DLBCL.

## Graphical abstract

Apatinib enhances the chemosensitivity of ABT‐199. Combined treatment inhibits tumour cell growth and promotes tumour cell death by regulating EDN1 and MAPK‐related pathways, and by activating the intrinsic apoptotic pathway.

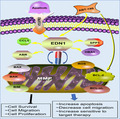

**Abbreviations**
CCK‐8: cell counting kit‐8
CI: combination index
CLL: chronic lymphocytic leukaemia
DLBCL: diffuse large B‐cell lymphoma
EDN1: endothelin‐1
GO: Gene Ontology
MMP: mitochondrial membrane potential
NC: negative control
NS: no significant
RNA‐seq: RNA deep‐sequencing
RNAseq: RNA sequencing
ROS: reactive oxygen species
siRNA: small‐interfering RNA
VEGFR‐2: vascular endothelial growth factor receptor‐2

## 1 Introduction

Diffuse large B‐cell lymphoma (DLBCL), the most common type of B‐cell lymphoid malignancies in adults, is a genetically heterogeneous disease. According to the cell of origin, it can be divided into two groups: germinal centre B‐cell‐like (GCB) and activated B‐cell‐like (ABC) subtypes [1, 2, 3, 4]. R‐CHOP (rituximab, cyclophosphamide, doxorubicin, vincristine and prednisone), a first‐line regimen, has a cure rate of more than 60% for DLBCL. The prognoses of patients who failed with R‐CHOP were often poor, especially those who are ineffective for frontline or subsequent therapies [5]. A new combined chemotherapy regimen is particularly important.

BCL‐2 is an important anti‐apoptotic protein, governing the intrinsic apoptotic pathway [6]. It is also highly expressed in 70% of DLBCL patients [7]. Overexpression of BCL‐2 leads to poor prognosis in patients with DLBCL after first‐line treatment [8, 9, 10, 11]. Venetoclax (ABT‐199), a small‐molecule inhibitor selectively targeting BCL‐2, has been approved by more than 50 countries for the treatment of adult chronic lymphocytic leukaemia (CLL) [12]. ABT‐199 has shown superior clinical activity in almost all cases of CLL [13]. In recent years, studies have shown that ABT‐199 has unlimited application potential in DLBCL and can significantly improve the survival rate and clinical symptoms of patients, which is gratifying for patients and researchers [14]. However, despite the progress of research, it is found that ABT‐199 leads to different degrees of drug resistance in DLBCL treatment, which also limits the application of ABT‐199 in DLBCL.

In the clinical treatment of leukaemia, the main way to overcome ABT‐199 resistance is to combine it with other chemotherapeutic drugs such as Azacytidine [15]. Apatinib (YN968D1), a small‐molecule tyrosine kinase receptor inhibitor selectively targeting VEGFR‐2 [16, 17], has been applied to patients with advanced gastric cancer in China [18]. Moreover, Apatinib has been used in phase II/III clinical trials of solid tumours (such as non‐small‐cell lung cancer, breast cancer, and hepatocellular carcinoma) [19, 20, 21]. For patients with advanced esophageal squamous cell carcinoma who failed to respond to first‐line chemotherapy drugs, the median progression‐free survival was 6.23 months, 2 (16.67%) patients achieved partial remission, and 9 (75.00%) achieved stable disease after they used Apatinib combined with S‐1regimen [22]. *In vitro* and phase I clinical studies have found the potential additive or synergistic antitumour effects between anti‐PD‐1 antibodies and VEGF/VEGFR‐2 inhibitors [23, 24], which prompted us to investigate whether Apatinib combined with ABT‐199 can exert a better therapeutic effect in DLBCL.

It has been found that the expression of VEGFR‐2 is affected by BCL‐2 [25]. Our previous research demonstrated that CS2164 (Chiauranib, targeting tumourigenesis‐associated pathways) combined with ABT‐199 improved the prognosis of B‐cell lymphoma [26]. However, compared with CS2164, Apatinib has higher antitumour activity in advanced cancers [27].

In this study, we sought to verify the potential synergistic antitumour effect of a regimen combining ABT‐199 with Apatinib in DLBCL. Administration of low‐dose ABT‐199 potentiates the cytotoxicity of Apatinib in various human DLBCL cell lines and primary DLBCL samples, as well as antitumour efficacy in xenograft mouse models. Mechanistically, ABT‐199 combined with Apatinib can synergistically kill DLBCL cells by regulating endothelin‐1 (EDN1) and related MAPK/ERK/MEK pathway to alter the balance of pro‐apoptotic vs. antiapoptotic BCL‐2 proteins without a significant increase in systemic toxicity.

## 2 Materials and methods

### 2.1 Drugs and reagents

ABT‐199 and Apatinib were purchased from Selleck Chemicals (Houston, TX, USA). The reagents were prepared as 10 mm stock solution in 100% dissolved in DMSO (Invitrogen, Carlsbad, CA, USA) stored at −20 °C, then diluted to the required concentrations according to the manufacturers' instructions.

### 2.2 Cell lines and cell culture

Human DLBCL cell lines OCI‐Ly19, OCI‐Ly1, OCI‐Ly3, OCI‐Ly10 and SU‐DHL‐4 were purchased from ATCC (Rockefeller, MD, USA) and DLBCL cell lines were grown in RPMI‐1640 medium (Gibco, Billings, MT, USA). OCI‐Ly3 cells were cultured in IMDM (Gibco). Supplemented with 10% fetal bovine serum (HyClone, Thermo Scientific, Waltham, MA, USA) at 37 °C in a humidified CO_2_ incubator. The primary DLBCL samples (*n* = 12) were obtained from the Department of Hematology, the First Affiliated Hospital, College of Medicine, Zhejiang University (Hangzhou, China) between 2021 and 2022. This study was approved by the Ethics Committee of the First Affiliated Hospital of Zhejiang University. The study methodologies conformed to the standards set by the Declaration of Helsinki and the experiments were undertaken with the understanding and written consent of each subject.

### 2.3 Cell viability assay

The cytotoxic effect of ABT‐199 and Apatinib on DLBCL cells was tested by cell counting kit‐8 (CCK‐8) assay (Dojindo, Kumamoto, Japan). In brief, cells (2 × 10^4^ cells per well) were seeded in 96‐well plates containing a 100 μL growth medium and treated with different concentrations of ABT‐199 or Apatinib alone or in combination for 12 or 24 h. CCK‐8 reagents (10 μL per well) were added and incubated for an additional 2–4 h at 37 °C, then the absorbance at 450 nm was detected by a microplate reader (ELx800; BioTek Instruments Inc., Winooski, VT, USA). Experiments were implemented in triplicate for each cell line. In the light microscopic experiment, cells were seeded in 24‐well plates and treated with different concentrations of ABT‐199 or Apatinib alone or in combination for 24 h. The results of cells were performed under a light microscope.

### 2.4 Flow cytometric assay for determining apoptosis, cell cycle, mitochondrial membrane potential and reactive oxygen species

To assess apoptosis, DLBCL cells were treated with different concentrations of ABT‐199 and Apatinib alone or in combination for 12 and 24 h, according to the manufacturer's instruction. DLBCL cells were harvested and then analysed by Novocyte (ACEA Bioscience, San Diego, CA, USA) after Annexin V/PI (Thermofisher, San Diego, CA, USA) staining for 15 min at room temperature in the dark. Primary DLBCL bone marrow or tissue samples were subjected to leukocyte separation. We used the Click‐iT EdU Kit (Thermofisher) to test the cell cycle. Loss of the mitochondrial membrane potential (MMP) (Δψm) was detected using JC‐1 Fluorescent Probe Kit (Beyotime Company, Shanghai, China). To evaluate the interaction between ABT‐199 and Apatinib, calcusyn software (Biosoft, Cambridge, UK) was used to calculate the combination index (CI). We used a Reactive Oxygen Species Assay Kit (Beyotime) as previously reported to detect the level of cellular reactive oxygen species (ROS), and the results were shown as the ratio of the mean fluorescence intensity.

### 2.5 Clonogenic assay

We used a colony‐forming assay to verify the inhibition effect of ABT‐199 and Apatinib alone or in combination, DLBCL cells were treated with different concentrations of agents, and then cells were planted in methylcellulose medium (Methocult H4100; Stem Cell Technologies, Vancouver, BC, Canada) at a density of 500 cells per well for about 14 days. The clone was stained with MTT and counted for tumour‐forming capability *in vivo*, and the size of the clonogenic was observed under a light microscope.

### 2.6 Western blot analysis

OCI‐Ly1 and OCI‐Ly19 cells were lysed at 4 °C in lysis buffer and were electrophoresed in 10% SDS/PAGE and then transferred to an NC membrane (Millipore, Billerica, MA, USA). The membranes were blocked for 1.5 h with 5% nonfat milk in TBS‐T and then probed with primary antibodies (1 : 1000 in 5% bovine serum albumin in TBS‐T) overnight at 4 °C according to the manufacturers, followed by secondary HRP‐conjugated antibody (1 : 20000; CST, Beverly, MA, USA) and visualised with an ECL detection kit (Biological Industries, Beit HaEmek, Israel). The primary antibody against EDN1 was purchased from Abcam (Cambridge, MA, USA). VEGFR‐2, p‐VEGFR‐2, ERK, p‐ERK, P38, p‐P38, MEK, p‐MEK, Caspase 3, PARP, BAX, BIM, BCL‐2, MCL‐1 and β‐actin were purchased from Cell Signaling Technology (Danvers, MA, USA).

### 2.7 RNA sequencing

Cells were incubated with single‐agent ABT‐199 (1 nm) or a combination of ABT‐199 and Apatinib (10 μm) for 24 h, then total RNA was extracted. RNA sequencing (RNAseq) was then carried out via a biological company (service ID# F20FTSECWLJ3511, BGI, Huada Gene, Wuhan, China). The heatmap was drawn by Pheatmap according to the gene expression in different groups. Essentially, differential expression analysis was performed using the deseq2 [28] with a *Q* value ≤ 0.05. Gene Ontology and KEGG enrichment analysis was performed by Phyper based on Hypergeometric test. The significant levels of terms and pathways were corrected by *Q* value with a rigorous threshold (*Q* value ≤ 0.05) by Bonferroni [29]. The pathway activity network was constructed using cytoscape [30] for graphical representations of enriched biological pathways with significance (*P* < 0.05). The computational method used to find the most important seed genes is MCODE.

### 2.8 *In vivo* study of Apatinib/ABT‐199 efficacy in DLBCL mouse models

Female immunodeficient BALB/C nude mice at 4–6 weeks of age were purchased from Zhejiang University Animal Center and acclimated for 1 week. After passing the quarantine, the animals were raised in a barrier environment without specific pathogen free in the laboratory animal centre of Zhejiang University. The mice were killed by cervical dislocation when the experiment is finished. All experiments were approved by the Ethics Committee for Laboratory Animals of the First Affiliated Hospital, College of Medicine, Zhejiang University (Hangzhou, China) (Reference number: 624) and were conducted following the National Institutes of Health Guide for the Care and Use of Laboratory Animals. For model #1, mice were injected subcutaneously with 2 × 10^7^ OCI‐Ly1 cells on the right flank mice. When the tumour volume reached ~ 75 mm^3^, mice were randomly divided into four groups (*n* = 5/group), including control, ABT‐199, Apatinib, and combination, and then the drugs were given for two consecutive weeks with control (vehicle, 0.2% methyl cellulose and 0.1% Tween‐80 in PBS), Apatinib (administered by oral gavage at the dose of 100 mg·kg^−1^·day^−1^), ABT‐199 (80 mg·kg^−1^·day^−1^, oral gavage), or combination of ABT‐199 and Apatinib, respectively. During the administration, the mice were monitored daily and their weight was recorded for toxicity. Two weeks after treatment, mice were killed by cervical dislocation and the tumour size was measured daily by caliper we calculated the volume (*V*) of the tumour using the equation: *V* = (*L* × *W* ^2^)/2, where *L* and *W* represent the length and width, respectively. The tumour tissue was subjected to immunohistochemical staining with human CD45 antibody and western blot analysis.

For model #2, after the knock‐down of the EDN1 gene, OCI‐Ly19 and OCI‐Ly1 cell line was injected subcutaneously into female BALB/C nude mice. Mice were monitored and weighed daily. After 2 weeks, subcutaneous tumours were stripped, and the maximal diameter (*L*) and short diameter (*W*) of the tumour were measured.

### 2.9 Gene overexpression and knock‐down

The open reading frame of human EDN1 cDNA was inserted into the lentiviral transfer vector pLV‐EF1a‐ IRES‐EGFP and verified the construction by Sanger sequencing. EDN1 was knocked down by lentiviral transduction using an EDN1‐specific shRNA transfer vector targeting residues 2494‐PGMLV‐SC5 (shEDN1‐1) and PGMLV‐6395 (shEDN1‐2) on RefSeq NM_001955.5. We used 293T to package the lentivirus and harvested it at 48 and 72 h. The target cells were transduced for two consecutive days and added 6 μg·mL^−1^ of polybrene. After 48 h of transfection, the fresh medium containing puromycin (1 μg·mL^−1^) was replaced. Western blot was used to detect the effect of overexpression and knock‐down.

### 2.10 Statistical analysis

All experiments were performed in triplicate when indicated, and the value was expressed as mean ± standard deviation (SD). graphpad prism 7 software (GraphPad Software, La Jolla, CA, USA) was used for statistical data. The Student's *t*‐test was used for mean comparison between the two groups. Multiple‐group comparisons were performed using the one‐way analysis of variance followed by the Bonferroni *post hoc* test. To verify the combination effect, we used calcusyn v2.0 software for calculating the CI. Synergism (CI < 1), addictive effect (CI = 1), or antagonism (CI > 1) [31]. *P* < 0.05 was considered statistically significant, and different levels were described as *P < 0.05, **P < 0.01, and ***P < 0.001, respectively. All statistical analyses were performed using spss 20.0 software (La Jolla, CA, USA).

## 3 Results

### 3.1 ABT‐199 cooperates with Apatinib to reduce the viability of diverse DLBCL cells

**Fig. 1:**
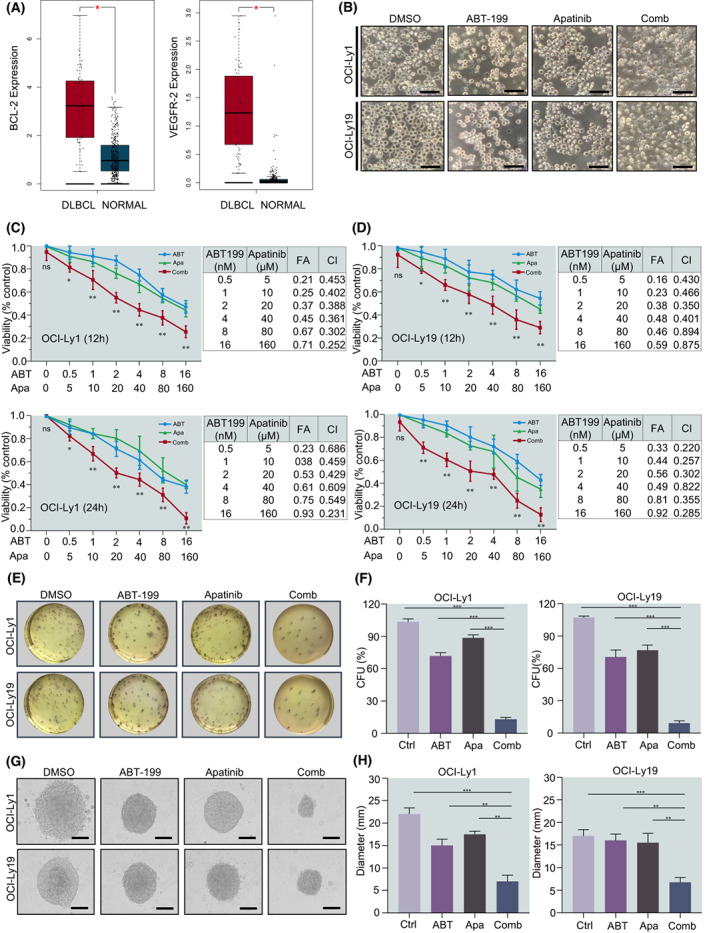
The BCL‐2 inhibitor ABT‐199 synergistically interacts with the VEGFR‐2 inhibitor Apatinib to inhibit cell viability in DLBCL cells. (A) The expression of BCL‐2 and VEGFR‐2 in DLBCL was higher than that in normal tissues. (B) Changes in morphology in OCI‐Ly1 and OCI‐Ly19 cells were incubated with ABT‐199 and Apatinib alone or combination treatment for 24 h and visualised using an inverted microscope. Scale bar: 20 μm. (C, D) The inhibition rate of cell viability was measured at 12 and 24 h in OCI‐Ly1 and OCI‐Ly19 cell lines using the CCK‐8. ns, no significant. (E, F) OCI‐Ly1 and OCI‐Ly19 cells were treated with ABT‐199 2 nm and Apatinib 20 μm for 24 h, after which the clonogenicity assay was performed to determine the percentage of CFU (E, representative images; F, bar graphs). (G, H) An inverted microscope observed the CFU size and measured its diameter (G, representative images; H, bar graphs) (scale bar: 20 μm). Data are presented as mean ± SEM. Statistical analyses were performed using unpaired Student's *t* tests. All experiments were repeated three times (*n* = 3). *P < 0.05, **P < 0.01, and ***P < 0.001.

In hematological malignancies, compared with the normal group, patients with DLBCL had high expression of BCL‐2 and VEGFR‐2 (Fig. 1A). To further explore new potential combination therapies, we used bioinformatics methods to analyse the signal pathways related to cell biological functions after ABT‐199 resistance, including cell cycle, apoptosis, cell migration and so on. This has important guiding significance for us to choose a new treatment regimen (Fig. S1A). To evaluate the potential cooperative effect of ABT‐199 and Apatinib, we assessed the effect on cell viability. Various DLBCL cell lines were treated with indicated concentrations of ABT‐199 and Apatinib, under an electron microscope we observed the cells' shrinkage, and the fragmentation of membrane blebs (Fig. 1B; Fig. S1B), after which cell viability was tested using CCK‐8 assay. Treatment with ABT‐199 and Apatinib alone effectively inhibited cell growth in a dose‐dependent manner. Notably, co‐administration of ABT‐199 and Apatinib led to markedly enhanced growth inhibition in all of these DLBCL cell lines (Fig. 1C,D; Fig. S1C–E). The half‐maximal inhibitory concentration (IC_50_) values (Table 1) of ABT‐199 and Apatinib were lower at 24 h than at 12 h in all cell lines (*P* < 0.001). Then, we examined whether the combination of ABT‐199 and Apatinib affects the clonogenicity of DLBCL cells (Fig. 1E,F; Fig. S2A,B). The size of colonies was observed under an electron microscope and visualisation. As shown in Fig. 1G,H and Fig. S2C,D the colony‐forming assay revealed that compared with ABT‐199 and Apatinib monotherapy groups the combination group could significantly inhibit colony formation in all tested cell lines (*P* < 0.001). Together, these results indicate that ABT‐199 synergistically interacts with Apatinib to reduce the viability of DLBCL cells.

**Table 1:** IC_50_ values of ABT‐199 and Apatinib as single agent in DLBCL cells. IC_50_, half‐maximal inhibitory concentration.

DLBCL cell lines	IC_50_ at 12 h		IC_50_ at 24 h	
	ABT‐199 (nm)	Apatinib (μm)	ABT‐199 (nm)	Apatinib (μm)
OCI‐Ly19	11.0 ± 1.23	4.6 ± 2.04	4.90 ± 1.61	2.80 ± 0.33
OCI‐Ly1	171 ± 1.46	16.2 ± 0.81	31.3 ± 1.82	2.04 ± 1.10
OCI‐Ly3	215 ± 1.72	36.1 ± 0.91	54.2 ± 1.33	15.2 ± 0.77
OCI‐Ly10	64.1 ± 0.52	23.1 ± 3.40	8.25 ± 1.74	5.92 ± 1.15
SU‐DHL‐4	26.1 ± 1.33	7.20 ± 2.11	13.3 ± 0.42	5.41 ± 0.53

### 3.2 Co‐exposure to ABT‐199 and Apatinib induces apoptosis of DLBCL cells, in association with altered cell cycle distribution, increased ROS generation, and mitochondrial injury

**Fig. 2:**
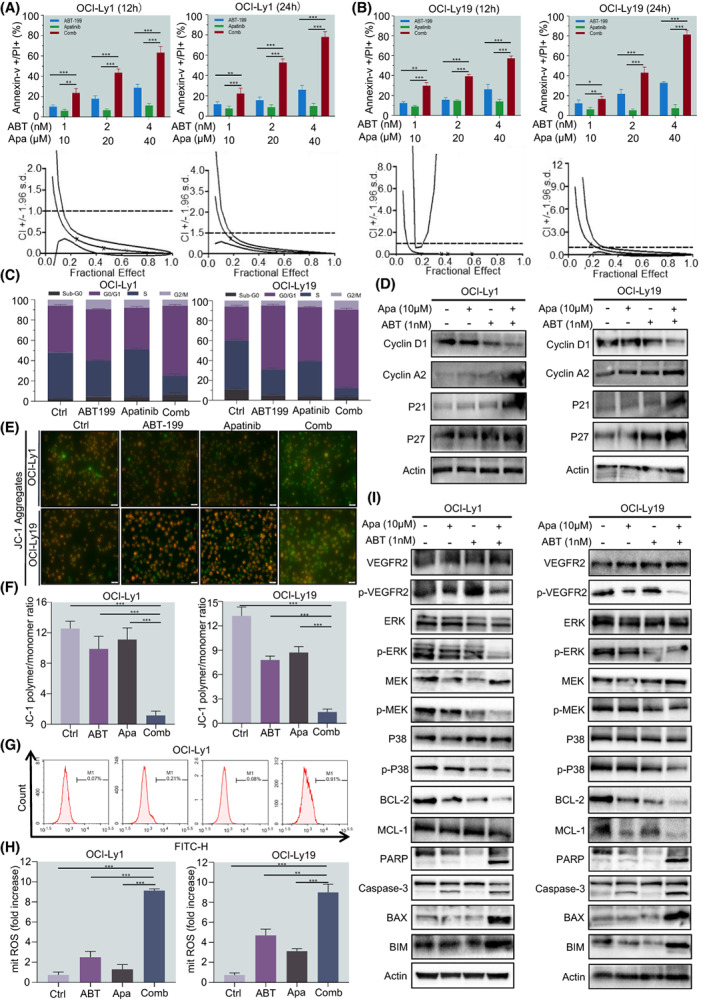
Co‐exposure to ABT‐199 and Apatinib induces apoptosis of DLBCL cells, in association with altered cell cycle distribution, increased ROS generation, and mitochondrial injury. (A, B) Cells were treated with the indicated concentrations of ABT‐199 ± Apatinib for 12 and 24 h, after which the percentage of Annexin‐V+ apoptotic cells was determined by flow cytometry after Annexin‐V and PI double staining. The CI was calculated based on apoptosis using the calcusyn software to evaluate the interaction between ABT‐199 and Apatinib in DLBCL cell lines (CI < 1.0 = 1.0, and > 1.0, indicating synergistic, additive, and antagonistic effect, respectively). (C) Cell cycle distribution was assessed at 24 h by flow cytometry (black, dark purple, black‐purple, and lavender present the statistical significance of Sub‐G0, G0/G1, S, G2/M between DMSO and Combo group, respectively). (D) Cell lysates after treatment with ABT‐199 and Apatinib alone or a combination were collected, and levels of cell cycle‐related proteins P21, P27, CyclinA2, and CyclinD1 were determined by western blotting with the respective antibodies. (E, F) OCI‐Ly1 and OCI‐Ly19 cells with loss of MMP (scale bar: 100 μm). (G, H) Meanwhile, intracellular ROS levels were measured by flow cytometry using the Reactive Oxygen Species Assay Kit. (I) OCI‐Ly1 and OCI‐Ly19 cells were exposed to the indicated concentrations of ABT‐199 ± Apatinib for 24 h, after which western blot analysis was shown to monitor the expression of p‐VEGFR‐2, MAPK pathway associated proteins, antiapoptotic proteins BCL‐2 and MCL‐1, as well as cleavage of caspase 3 and PARP. Data are presented as mean ± SEM. Statistical analyses were performed using unpaired Student's *t* tests. All experiments were repeated three times (*n* = 3). *P < 0.05, **P < 0.01, and ***P < 0.001.

To further verify the cytotoxicity effect of ABT‐199 and Apatinib when used in combination, we treated DLBCL cells with ABT‐199 at various concentrations (1, 2, 4 nm) in the presence or absence of different concentrations of Apatinib (10, 20, 40 μm) for 12 and 24 h and then the percentage of apoptotic cells was detected by Annexin V/DAPI dual staining. The results showed that, even though exposure to different concentrations of ABT‐199 and Apatinib single treatment can induce a certain degree of apoptosis, these events were significantly enhanced by combined treatment for 12 and 24 h (Fig. 2A,B; Fig. S3D–F). The CI calculation shows that ABT‐199 has a strong synergistic effect with Apatinib at different doses (Table 2). It is well accepted that caspase activation plays an important role in the execution of apoptosis. Thus, we wonder whether blocking the activation of caspase by Z‐VAD‐fmk (pan‐caspase inhibitor) could rescue the apoptosis induced by the combinatorial treatments in DLBCL cells. Apoptotic status was analysed after 24 h of exposure to ABT‐199/Apatinib/the combination in the presence or absence of Z‐VAD‐fmk (20 μm). As shown in Fig. S3G, treatment with Z‐VAD‐fmk tremendously rescued apoptosis induced by the combination of ABT‐199 and Apatinib treatment. Subsequently, we analysed the cell cycle status to further characterise the role of low‐dose ABT‐199 in enhancing Apatinib‐mediated cytotoxicity. In all tested DLBCL cell lines, compared with the ABT‐199 or Apatinib alone, the percentage of S phase cells in the combined group decreased significantly after 24‐h treatment, and the cell cycle was arrested in G0/G1 phase (Fig. 2C; Fig. S3A–C). In addition, ABT‐199 combined with Apatinib also regulated cycle‐related proteins (Fig. 2D). Consistent with the results of apoptosis, ABT‐199 and Apatinib also induced loss of MMP, reflected by markedly decreased fluorescence intensity ratio between JC‐1 aggregate and monomer (Fig. 2E,F). To uncover the potential mechanism of apoptosis induced by these two agents, flow cytometry was used to detect intracellular ROS levels. After being cotreated with ABT‐199 and Apatinib for 24 h, a notable increase in ROS generation was observed in OCI‐Ly1 and OCI‐Ly19 (Fig. 2G,H), compared with treatment with every single agent. In addition, our results showed that ABT‐199 and Apatinib exerted an antiproliferative effect in DLBCL cells by inhibiting the MAPK/ERK and pro‐survival pathways in OCI‐Ly1 and OCI‐Ly19 cell lines (Fig. 2I). Together, these results suggest that ABT‐199 combined with Apatinib induces apoptosis, and alters the cell cycle distribution of DLBCL cells via promotion of ROS production and mitochondrial damage.

**Table 2:** Combination index value of ABT‐199 combined with Apatinib in DLBCL cell lines. ED50, median effective dose.

DLBCL cell lines	Combination index at 12 h			Combination index at 24 h		
	ED50	ED75	ED90	ED50	ED75	ED90
OCI‐Ly19	0.02332	0.01065	0.00494	0.19161	0.04673	0.01201
OCI‐Ly1	0.11521	0.04058	0.01439	0.15799	0.05508	0.01937
OCI‐Ly3	0.05283	0.01781	0.00632	0.03605	0.00444	0.00058
OCI‐Ly10	0.11342	0.03869	0.01332	0.10958	0.03948	0.01446
SU‐DHL‐4	0.00721	0.00164	0.00086	0.02949	0.00794	0.00216

### 3.3 The regimen combining ABT‐199 and Apatinib impairs tumour‐forming capabilities in a xenograft model

**Fig. 3:**
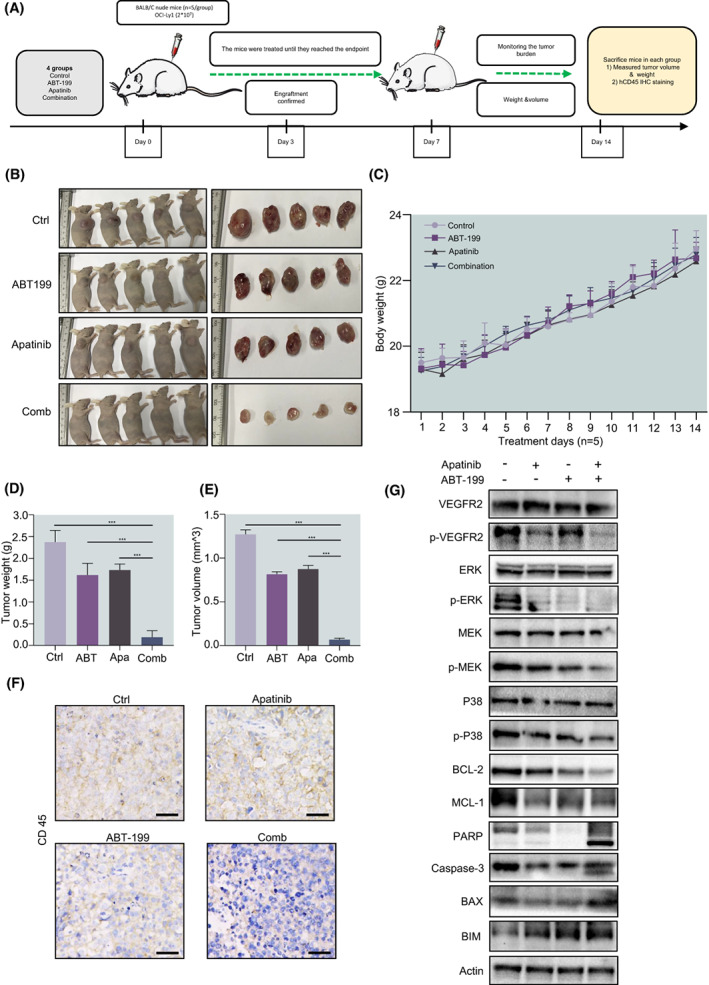
BALB/C nude mice were injected subcutaneously with 2 × 10^7^ OCI‐Ly1 cells at the right flank. Once the tumour volume reached to ~ 75 mm^3^, mice were randomly divided into four groups (*n* = 5/group) including control (Ctrl), ABT‐199, Apatinib and combination (Comb), then treated for 14 consecutive days with vehicle (0.2% methyl cellulose and 0.1% Tween‐80 in PBS), ABT‐199 (80 mg·kg^−1^·day^−1^, oral gavage), Apatinib (100 mg·kg^−1^·day^−1^, oral gavage), or combined ABT‐199 and Apatinib, respectively. (A) Implementation scheme of *in vivo* experiment. (B) Tumour size. (C) During treatment, the bodyweight of mice was monitored daily. (D, E) Tumour weight and volume. (F) Immunohistochemical staining for human CD45 was performed to examine the infiltration of tumour cells (scale bar: 20 μm). (G) Western blot analysis for p‐VEGFR‐2, MAPK pathway associated proteins, BCL‐2, and MCL‐1 on tumour homogenate. All western blot experiments were repeated three times (*n* = 3). Data are presented as mean ± SEM. Statistical analyses were performed using unpaired Student's *t* tests. ***P < 0.001.

To confirm the therapeutic effect of BCL‐2 inhibitor combined with Apatinib on DLBCL *in vivo*, we tested the efficacy of ABT‐199 plus Apatinib in the BALB/C nude mice (Fig. 3A). The DLBCL cell line OCL‐Ly1 was subcutaneously injected for about 7 days, and the treatment was started when the tumour volume reached ~ 75 mm^3^, mice were randomly divided into the vehicle, ABT‐199 (80 mg·kg^−1^), Apatinib (100 mg·kg^−1^), and the combination of ABT‐199 plus Apatinib groups for 14 consecutive days, respectively. Interestingly, compared with the vehicle control as well as each agent, the combined group significantly reduced tumour burden, reflected by decreasing the volume and weight of the tumour (Fig. 3B,D,E). In addition, the weight of the mice did not decrease significantly and no other signs of notable toxicity were observed (Fig. 3C). Histological examination showed that the combination treatment inhibited the infiltration of DLBCL cells, whilst diseased mice treated with ABT‐199 or Apatinib alone had more severe DLBCL infiltration (Fig. 3F). Western blot revealed that the MAPK/ERK pathway was inhibited, which is consistent with the results *in vitro* (Fig. 3G). Together, these findings argue that ABT‐199 and Apatinib might interact to cause robust apoptosis and inhibition of DLBCL cells by downregulation of MAPK/ERK pathway and anti‐apoptotic BCL‐2 family proteins as well as upregulation of pro‐apoptotic BAX in DLBCL cells. Together, these findings indicate that the combination regimen of ABT‐199 and Apatinib is effective *in vivo* against DLBCL, whilst well tolerated.

### 3.4 Combined treatment with ABT‐199 and Apatinib ameliorates DLBCL primary patient cells *in vitro* and *in vivo*

**Fig. 4:**
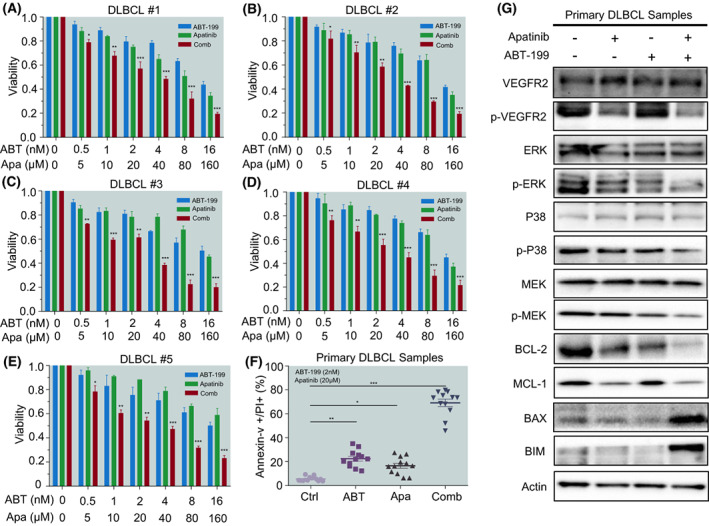
ABT‐199 cooperates with Apatinib in killing primary DLBCL cells. Primary bone marrow cells and lymph nodes isolated from 12 DLBCL patients were treated with different concentrations of ABT‐199 and Apatinib alone or in combination for 24 h. (A–E) Cell viability was measured by the CCK8 assay. Data are presented as mean ± SD; *P* values were calculated by the comparison between the combination group and ABT‐199 or Apatinib alone group. (F) Primary blast cells were exposed to the indicated concentrations of ABT‐199 or Apatinib alone or in combination for 24 h, after which the percentage of Annexin‐V+ apoptotic cells was determined by flow cytometry after Annexin‐V and PI double staining. (G) Western Blot analysis for MAPK pathway associated proteins, BCL‐2, and MCL‐1 from primary bone marrow cells and lymph node homogenate. All western blot experiments were repeated three times (*n* = 3). Data are presented as mean ± SEM. Statistical analyses were performed using unpaired Student's *t* tests. *P < 0.05, **P < 0.01, and ***P < 0.001.

The antitumour effect of ABT‐199 and Apatinib alone or in combination was further verified *ex vivo* in the primary blasts isolated from 12 DLBCL patients. We summarised the characteristics of the patients in Table 3. Following the results obtained from DLBCL cell lines, cotreatment of primary DLBCL blasts with ABT‐199 at concentrations ranging from 0.5 to 16 nmol·L^−1^ and Apatinib from 5 to 160 μmol·L^−1^ caused remarkable inhibited cells proliferation when compared with each agent alone (Fig. 4A–E). We detected the apoptosis of primary DLBCL blasts and found that ABT‐199 and Apatinib could significantly induce apoptosis, which was consistent with the results in cell lines (Fig. 4F). Similarly, western blot results showed that MAPK/ERK pathway and anti‐apoptotic BCL‐2, and MCL‐1 were significantly inhibited (Fig. 4G). Together, these results indicate that the combination of ABT‐199 with Apatinib might be a preferable choice for targeted DLBCL blasts. Together, the combination regimen of ABT‐199 with Apatinib is effective against primary DLBCL cells.

**Table 3:** Characteristic of primary DLBCL patients.

Patients	Diagnose	Gender	Age (year)	Karyo‐type	Stages	Origins	Immunohistochemistry
DLBCL#1	*De novo*	F	60	46, XX	IV/A	Non‐GCB	CD20(+)/CD19(+)/CD22(+)/CD30(+)/BCL6(+)/MUM1(+)/BCL2(+)/c‐MYC(+)
DLBCL#2	*De novo*	M	79	46, XY	III/A	Non‐GCB	CD20(+)/CD79a(+)/c‐MYC(+)/Ki‐67(+)/MPO(+)
DLBCL#3	*De novo*	M	66	46, XY	I/A	GCB	CD20(+)/CD19(+)/CD10(+)/CD30(+)/BCL6(+)/BCL2(+)/c‐MYC(+)/Ki‐67(+)
DLBCL#4	Refractory	M	83	46, XY	IV/A	Non‐GCB	CD20(+)/PAX‐5(+)/BCL6(+)/CD5(+)/MUM1(+)/c‐MYC(+)/Ki‐67(+)
DLBCL#5	*De novo*	M	67	46, XY	IV/B	Non‐GCB	CD20(+)/CD19(+)/PAX‐5(+)/BCL6(+)/MUM1(+)/BCL2(+)/c‐MYC(+)/Ki‐67(+)
DLBCL#6	*De novo*	M	80	46, XY	IV/B	GCB	CD20(+)/CD19(+)/CD10(+)/BCL6(+)/MUM1(+)/CD5(+)/CD21(+)/BCL2(+)
DLBCL#7	*De novo*	M	71	46, XY	IV/A	Non‐GCB	CD20(+)/CD19(+)/CD21(+)/CD79a(+)/CD70(+)/PAX‐5(+)/BCL6(+)/MUM1(+)
DLBCL#8	Refractory	F	65	46, XX	III/A	GCB	CD20(+)/CD19(+)/CD10(+)/PAX‐5(+)/BCL6(+)/MUM1(+)/CD30(+)/BCL2(+)
DLBCL#9	*De novo*	F	60	46, XX	IV/A	Non‐GCB	CD20(+)/CD19(+)/CD(+)/CD21(+)/CD23(+)/CD30(+)/BCL6(+)/MUM1(+)
DLBCL#10	Refractory	F	76	46, XX	IV/A	Non‐GCB	CD20(+)/CD79a(+)/Ki‐67(+)/CD3(+)/CD5(+)
DLBCL#11	Refractory	F	66	46, XX	II/A	Non‐GCB	CD20(+)/CD19(+)/PAX‐5(+)//BCL2(+)/c‐MYC(+)/BCL6(+)/CD22(+)/P53(+)
DLBCL#12	*De novo*	M	87	46, XY	IV/A	GCB	CD20(+)/CD79a(+)/CD10(+)/BCL2(+)/BCL6(+)/c‐MYC(+)/Ki‐67(+)

### 3.5 Cotreatment with ABT‐199 and Apatinib alters genome‐wide gene expression in DLBCL cells

**Fig. 5:**
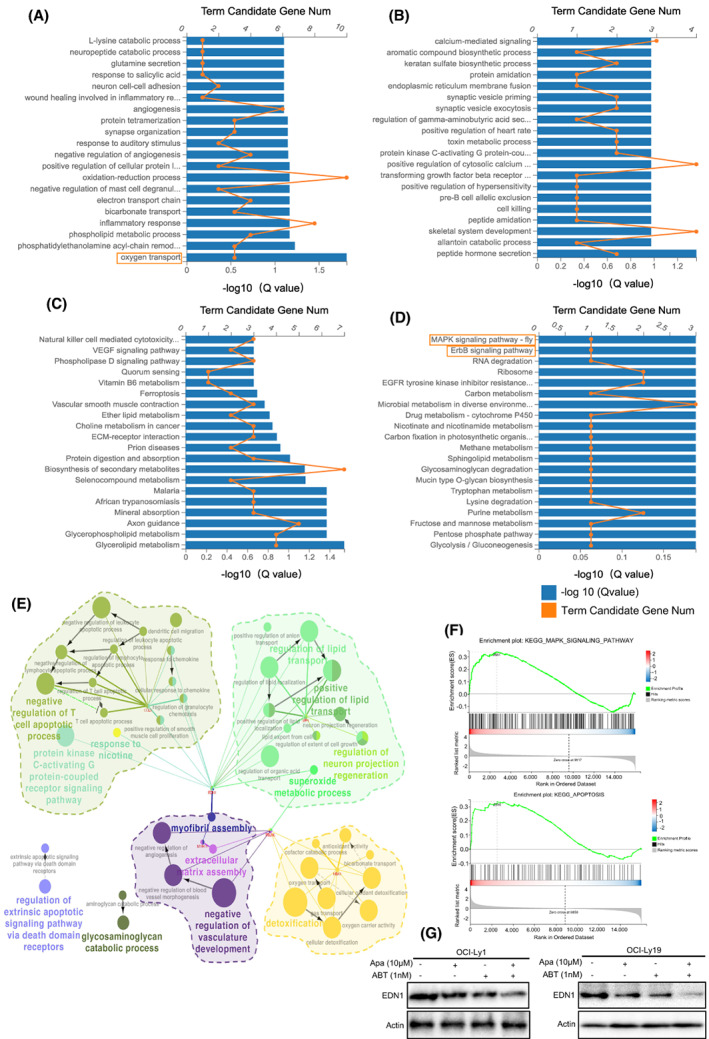
DLBCL cells transcriptome determined by RNAseq. (A, B) Gene Ontology (GO) analysis for differentially expressed genes treated with ABT‐199 or Apatinib for 24 h (A UP genes, B DOWN genes). (C, D) Pathway analysis for differentially expressed genes from the DLBCL cells transcriptome (C UP genes, D DOWN genes). The orange line means Term Candidate Gene Num and the blue bars mean −log10 (*Q* value). (E) Five KEY genes and GO activity network construction using cytoscape (*P* < 0.05). (F) GESA analysis of combination group using cytoscape (*P* < 0.05). (G) Western blot analysis was performed to validate the downregulation efficiency of Apatinib and ABT‐199 on EDN1 in OCI‐Ly1 and OCI‐Ly19 cells. All western blot experiments were repeated three times (*n* = 3).

To screen for the molecular target of cotreatment with ABT‐199 and Apatinib, we performed RNAseq in the samples of DLBCL cells treated with ABT‐199 and/or Apatinib. We found that 189 genes were differentially expressed after cotreatment ABT‐199 and Apatinib compared with that in the single‐treated samples (Table S1). GO analysis indicated that multicellular organismal development was upregulated, whilst terms including oxidation–reduction process, inflammatory response, oxygen transport and cell killing were downregulated (Fig. 5A,B). Pathway analysis revealed that natural killer cell‐mediated cytotoxicity, VEGF signalling pathway, and ECM‐receptor interaction were significantly elevated. Meanwhile, the MAPK signalling pathway and ErbB signalling pathway were downregulated (Fig. 5C,D). Five key genes including HGF, EDNZ, SPP1, HMOX1, CCL5 and ADM were significantly enriched according to the pathway activity network (Fig. 5E,F). Different K‐cores were used to identify core regulatory genes involved in DLBCL treatment. As shown in Fig. S4C, we found that EDN1 was most possibly related to DLBCL development, which achieved a high ranking amongst the top 5 genes in terms of different K‐cores after excluding genes with low abundance and matrix‐related genes. It was verified by western blot that Apatinib combined with ABT‐199 could effectively suppress EDN1 (Fig. 5G). Thus, we hypothesised that cotreatment exhibited its attenuation effect on DLBCL by down‐regulating EDN1. GO analysis was used to analyse differentially expressed genes treated with ABT‐199 and/or Apatinib for 24 h (all DEGs) and the percentage of GO terms in the combination treatment group is shown in the pie chart from the DLBCL cells transcriptome (all DEGs) (Fig. S4A,B). Together, these results suggest that the mechanism of action underlying the antitumour activity of the combination treatment with ABT‐199 and Apatinib might be through the regulation of the EDN1 gene.

### 3.6 Endothelin‐1 gene plays an important role in apoptosis induced by ABT‐199 and Apatinib

**Fig. 6:**
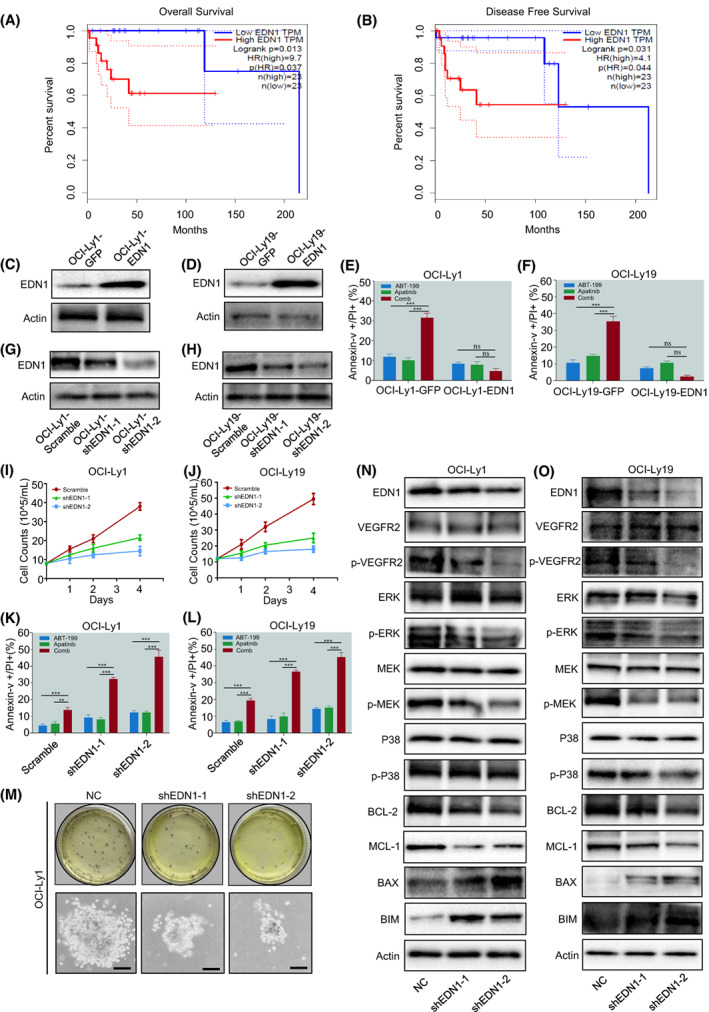
ABT‐199/Apatinib reduced viability and induced apoptosis of DLBCL cells by downregulation of EDN1. (A, B) EDN1 is a vital oncogene affecting overall survival (OS) and disease‐free survival (DFS). (C, D) EDN1 was overexpressing in OCI‐Ly1 cells (left) and OCI‐Ly19 (right). (E, F) OCI‐Ly1 and OCI‐Ly19 cells were treated with indicated concentrations of ABT‐199 ± Apatinib for 24 h, after which the percentage of apoptosis was determined by flow cytometry. (G, H) Levels of EDN1 in OCI‐Ly1 and OCI‐Ly19 cells transduced with lentivirus vectors containing control shRNA (NC), shRNA targeting EDN1 (shEDN1‐1), or shEDN1‐2 were detected by western blot analysis with antibodies indicated. (I, J) The proliferation of OCI‐Ly1 and OCI‐Ly19 cells expressing control and EDN1 shRNA were shown by counting viable cells for about 3 days. (K, L) OCI‐Ly1 and OCI‐Ly19 cells were treated with indicated concentrations of ABT‐199 ± Apatinib for 24 h, after which the percentage of apoptosis was determined by flow cytometry. (M) When the EDN1 is knocked down the clonogenicity assay was performed in OCI‐Ly1 cells to determine the percentage of CFU (scale bar: 20 μm). (N, O) Levels of EDN1, p‐VEGFR‐2, MAPK pathway associated proteins, anti‐apoptosis BCL‐2 and MCL‐1 were detected by western blot analysis. Actin was used as a loading control after EDN1 was knocked down. Data are presented as mean ± SEM. Statistical analyses were performed using unpaired Student's *t* tests. ns, no significant. All experiments were repeated three times (*n* = 3). **P < 0.01 and ***P < 0.001.

EDN1 is considered to be the most effective vasoconstrictor in the human cardiovascular system and plays a vital role in the development of tumours. In the previous study, we found the EDN1 gene through RNAseq assay, and it was also an anti‐apoptotic protein that affects overall survival and disease‐free survival (Fig. 6A,B). Therefore, we verified its role in DLBCL by overexpression and knock‐down of the EDN1 gene. Western blot and flow cytometry showed the effect of overexpression of the EDN1 gene in OCI‐Ly1 and OCI‐Ly19 cell lines (Fig. 6C–F; Fig. S2E,F). Then, we knocked down the EDN1 gene, and western blot was used to verify the knock‐down effect of EDN1 (Fig. 6G,H). Compared with the scramble, the expression of the EDN1 significantly decreased. The cell counts and colony‐forming also obviously decreased after shRNA interference (Fig. 6I,J,M; Fig. S2G). The flow cytometry results also showed that the proportion of apoptosis of OCI‐Ly1 and OCI‐Ly19 cell lines after interference was remarkably increased as shown in Fig. 6K,L. After the EDN1 gene was knocked down, the MAPK pathway was significantly inhibited (Fig. 6N,O). In addition, the apoptosis ratio of DLBCL cells was verified by flow cytometry after EDN1 was knocked down. Meanwhile, the EDN1 knock‐down cell lines were treated with ABT‐199 and Apatinib. The results showed that the proportion of apoptosis cells increased after EDN1 knock‐down compared with the control group. Moreover, the combination of the two drugs could further enhance this effect (Fig. S3H). Together, these results suggest that EDN1, an important oncogene, plays a role in the MAPK pathway.

### 3.7 shRNA interference of EDN1 gene inhibits tumour burden in mice

**Fig. 7:**
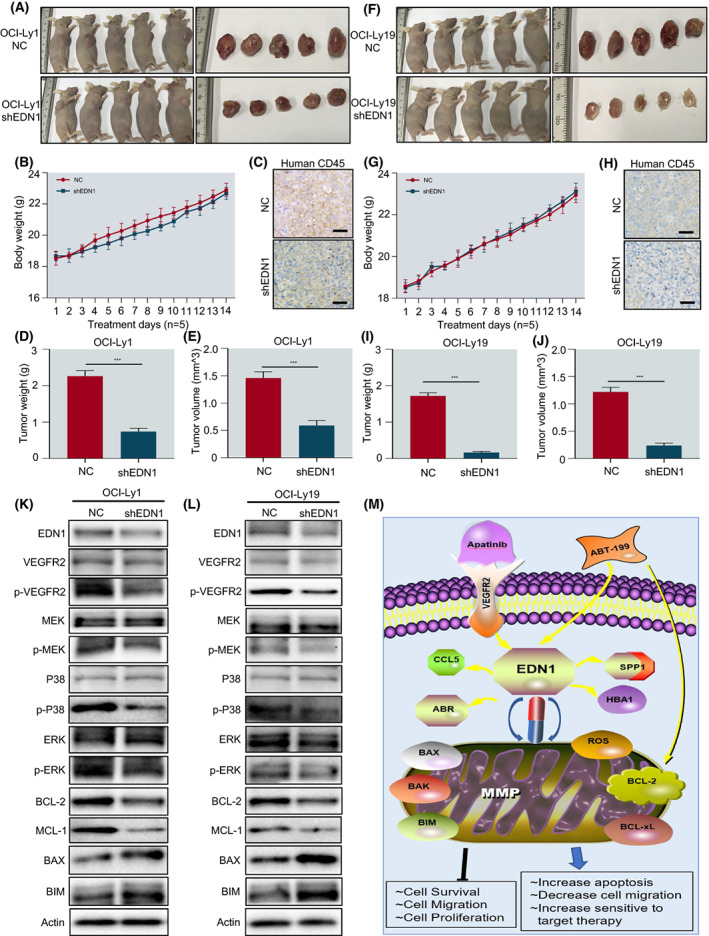
EDN1 knock‐down significantly reduced the tumour burden of BALB/C nude mice. (A, F) After the EDN1 was knocked down BALB/C nude mice were re‐injected with OCI‐Ly1 and OCI‐Ly19 cells (*n* = 5/group). The ruler (left margin) indicates size in millimeter. Compared with the negative control (NC), the tumour burden of mice was inhibited after being knocked down of EDN1. (B, G) During treatment, the bodyweight of mice was monitored daily. (D, E, I, J) Tumour weight and volume. (C, H) Immunohistochemical staining for human CD45 was used to examine the infiltration of tumour cells (scale bar: 20 μm). (K, L) Western blot analysis for EDN1, p‐VEGFR‐2, MAPK pathway associated proteins, BCL‐2, and MCL‐1 on tumour homogenate. (M) Pharmacologic targeting EDN1 induces mitochondrial dysfunction, decreased intracellular ROS levels, and apoptosis in DLBCL. All western blot experiments were repeated three times (*n* = 3). Data are presented as mean ± SEM. Statistical analyses were performed using unpaired Student's *t* tests. ***P < 0.001.

To further explore the effect of EDN1 knock‐down *in vivo*, we selected BALB/C nude model mice and injected EDN1 knock‐down OCI‐Ly1 and OCI‐Ly19 cell lines into mice respectively. According to the results of cell lines, EDN1 gene knock‐down can effectively inhibit the growth of tumour cells and reduce tumour load, reflected by decreased volume and weight of tumour masses, compared to vehicle control and each agent (Fig. 7A,D–F,I,J). In addition, there was no significant difference in body weight between the negative control (NC) group and the shEDN1 group (Fig. 7B,G). Histological examination found that the shEDN1 group remarkably inhibited the infiltration of DLBCL cells (Fig. 7C,H). After the mice were killed, we ground the stripped tumour and extracted proteins, and the results are consistent with those of cell lines. ABT‐199 and Apatinib inhibited the MAPK/ERK and pro‐survival pathways in the xenograft model (Fig. 7K,L). Thus, a mechanism involving ABT‐199 and Apatinib apoptotic networks is presented in hematologic malignancies (Fig. 7M). Together, after EDN1 was knocked down, the tumour load of mice was significantly reduced.

## 4 Discussion

Although the first‐line treatment, R‐CHOP (rituximab, cyclophosphamide, doxorubicin, vincristine, and prednisolone) and other conventional therapies combining multiple chemotherapeutic drugs have greatly improved the survival of many patients with DLBCL, this disease remains incurable [32, 33, 34, 35]. Therefore, new agents and drug combinations with less systemic toxicity are urgently needed. ABT‐199, a selective targeting inhibitor of BCL‐2, has emerged as a promising treatment strategy for some B‐cell malignancies such as CLL and MCL (mantle cell lymphoma). However, the therapeutic effect of ABT‐199 alone in DLBCL is limited [36]. In this study, we found a regimen combining BCL‐2 inhibitor ABT‐199 with tyrosine kinase inhibitor Apatinib exerting superb antitumour activity against DLBCL cells and patients, and this combination regimen showed superior effects on cells from different DLBCL subtypes carrying diverse genetic alterations in the *in vitro*, *ex vivo*. In addition, we observed that ABT‐199 combined with Apatinib was highly active in a BALB/C nude mouse model indicating the promising activity of this regimen for the treatment of high‐risk DLBCL patients with poor prognosis. Our previous research confirmed that CS2164 combined with ABT‐199 improved the prognosis of B‐cell lymphoma. Compared with CS2164 and ABT‐199, Apatinib and ABT‐199 have a higher antitumour activity for advanced cancers. In the treatment of B‐cell lymphoma, the IC_50_ value of ABT‐199 combined with Apatinib is lower than that of CS2164. Thus, the regimen combining ABT‐199 and Apatinib may represent an effective regimen to treat DLBCL.

Angiogenesis plays an important role in the development and metastasis of tumours. It is also an essential part of the growth of tumour cells [37, 38, 39]. Tumour angiogenesis is related to the activation of VEGF by binding to its specific receptor (VEGFR). Notably, the overexpression of VEGF and VEGFR correlates with the metastasis and microvessel density of many kinds of malignancies, so the prognosis is poor [40]. Therefore, VEGFR is a promising target for tumour therapy. Researchers reported that VEGF was also shown to enhance endothelial cell survival by upregulating BCL‐2 expression through a pathway mediated by VEGFR‐2 and phosphatidylinositol 3‐kinase/Akt signalling [41, 42]. In this paper, we verified that exposure to Apatinib resulted in marked p‐VEGFR‐2 and BCL‐2 downregulation, and BAX upregulation. This reveals that the expression of VEGFR‐2 will cause the expression of the anti‐apoptotic protein BCL‐2. Therefore, simultaneous inhibition of VEGFR‐2 and BCL‐2 can effectively inhibit the growth of DLBCL cells. Moreover, our RNAseq results showed that ABT‐199 combined with Apatinib‐regulated EDN1 gene, and EDN1 exerted antiproliferative effects by inhibiting the MAPK/ERK and pro‐survival pathways (Figs 5 and 6). Thus, these findings support the notion that EDN1 may play an important role in the combination of ABT‐199/Apatinib. They also propose a potential mechanism for the interaction between ABT‐199 and Apatinib, in which whilst ABT‐199 inhibits BCL‐2 expression, accompanied by Apatinib decreases p‐VEGFR‐2 expression.

Aberrant activation of the EDN1 axis is now generally considered a common mechanism underlying the progression of various solid tumours, including ovarian, prostate, colon, breast, bladder, and lung cancers, which is an important adverse factor affecting the prognosis [43, 44, 45]. Besides, the function of the EDN1 gene is related to the activation of the MAPK pathway [45]. In this study, we found that cotreatment with ABT‐199 and Apatinib resulted in marked EDN1 inhibition in DLBCL cells. On the contrary, ABT‐199 or Apatinib alone could not inhibit the expression of EDN1. In words, the killing effect of the same concentration of Apatinib and ABT‐199 on cells is weakened when EDN1 is highly expressed. EDN1 is an adverse gene and proper knock‐down enhances the therapeutic effect of Apatinib and ABT‐199. In addition, we used flow cytometry to verify the apoptosis ratio of DLBCL cells after EDN1 was knock‐down. Meanwhile, we added ABT‐199 and Apatinib to the knock‐down cell lines. The results showed that the proportion of apoptosis cells increased after EDN1 knock‐down compared with the control group. Moreover, the combination of the two drugs could further enhance this effect (Fig. S3H).

These observations proved that the EDN1 gene may play a crucial role in regulating the synergistic killing effect of DLBCL by ABT‐199 and Apatinib (Figs 5 and 6).

However, other genes and pathways that we have not studied may also play a role in this process. Anyway, this is also a defect of our experiment, which will be further improved in the follow‐up research.

The bulk of research on ABT‐199 and Apatinib alone in DLBCL has been widely published, especially ABT‐199, but the research combining ABT‐199 and Apatinib for the treatment of DLBCL has not been studied so far. In this context, our study found that ABT‐199/Apatinib was also effective on OCI‐Ly1, OCI‐Ly3 and OCI‐Ly10 cell lines from activated B‐cell‐like (ABC) which expression of BCL‐6, CD10, and MUM1, as well as primary DLBCL blasts from patients [46]. In addition, the efficacy of this regimen was further replicated in BALB/C nude mouse model. Moreover, the weight change and mental state of experimental mice showed that the toxicity and side effects of this regimen were small. Taken together, our results strongly suggest that the combination of ABT‐199 and the Apatinib regimen might represent an effective therapy for the treatment of DLBCL patients, and future studies are warranted to include more PDX models.

Strikingly, our regimen is effective not only for patients with primary DLBCL but also for refractory/relapsed patients, implying that the combination regimen could potentially diminish tumour burden in DLBCL patients. In addition, we also found that the combined regimen did no effect on peripheral WBC of healthy people, indicating that the regimen had less toxic side effects on DLBCL patients. It might also provide a theoretical basis for the clinical application of the combined regimen, although it needs to be further examined.

## 5 Conclusions

Our study provides strong preclinical evidence to support that the regimen combining ABT‐199 and Apatinib is highly effective towards DLBCL with diverse cytogenetic and genetic aberrations, including refractory/relapsed patients. The novel combination regimen still needs further clinical investigation in the treatment of DLBCL patients, especially those with multiple gene mutations.

## Conflict of interest

The authors declare no conflict of interest.

## Author contributions

YS and JY performed the experiment and drafted the manuscript. HS and YX conducted the patient samples data collection and analysis. RW participated in cell survival and migration assay. XY, JJ and WX conceived the research and finalised the manuscript. All authors have approved the final version of the manuscript and agree to be accountable for all aspects of the work.

## Supporting information


**Fig. S1.** Apatinib enhances chemosensitivity of ABT‐199 to reduce the viability of diverse DLBCL cells.

**Fig. S2.** Co‐exposure to ABT‐199 and Apatinib inhibit cell proliferation in DLBCL cells.

**Fig. S3.** ABT‐199 combined with Apatinib induces apoptosis of DLBCL cells.

**Fig. S4.** The percentage of GO terms in the ABT‐199 and/or Apatinib (all DEGs) treatment group is shown in the pie chart from the DLBCL cells transcriptome (all DEGs).

**Table S1.** Compared with the single‐treated samples, co‐treatment ABT‐199 and Apatinib changed 189 genes.

Legends

## Data Availability

The data sets used and/or analysed during the current study are available from the corresponding author on reasonable request.
