# Supplementary material for: Apatinib enhances chemosensitivity of ABT‐199 in diffuse large B‐cell lymphoma
Source: Mol Oncol. 2022 Sep 7;16(20):3735–53. doi: 10.1002/1878-0261.13309 (PMC9580892; doi:10.1002/1878-0261.13309)
Supplement: Supplementary file 1 — Fig. S1. Apatinib enhances chemosensitivity of ABT‐199 to reduce the viability of diverse DLBCL cells. [file MOL2-16-3735-s004.pdf]

A

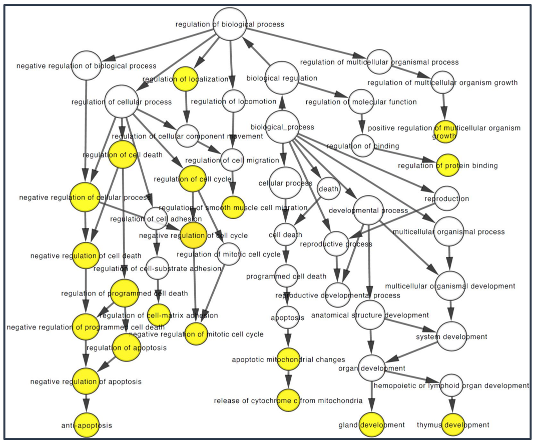


B

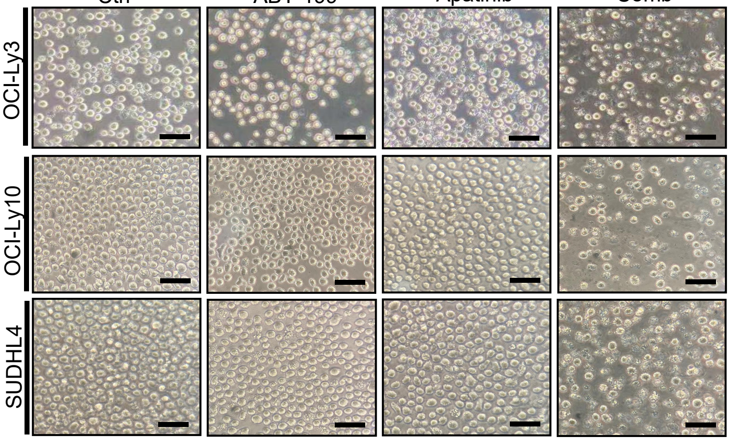


C

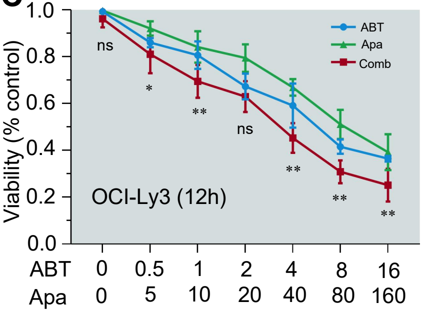


| ABT199 (nM) | Apatinib (μM) | FA | CI |
|---|---|---|---|
| 0.5 | 5 | 0.24 | 0.567 |
| 1 | 10 | 0.36 | 0.539 |
| 2 | 20 | 0.37 | 1.020 |
| 4 | 40 | 0.45 | 0.479 |
| 8 | 80 | 0.73 | 0.589 |
| 16 | 160 | 0.74 | 0.512 |

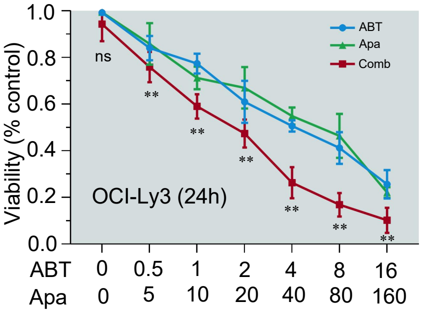


| ABT199 (nM) | Apatinib (μM) | FA | CI |
|---|---|---|---|
| 0.5 | 5 | 0.27 | 0.587 |
| 1 | 10 | 0.46 | 0.349 |
| 2 | 20 | 0.57 | 0.371 |
| 4 | 40 | 0.79 | 0.172 |
| 8 | 80 | 0.87 | 0.156 |
| 16 | 160 | 0.93 | 0.122 |

D

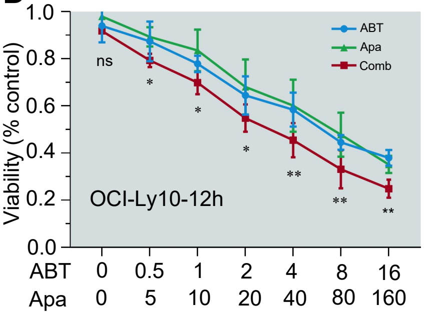


| ABT199 (nM) | Apatinib (μM) | FA | CI |
|---|---|---|---|
| 0.5 | 5 | 0.22 | 0.516 |
| 1 | 10 | 0.33 | 0.576 |
| 2 | 20 | 0.49 | 0.582 |
| 4 | 40 | 0.59 | 0.778 |
| 8 | 80 | 0.72 | 0.883 |
| 16 | 160 | 0.79 | 1.227 |

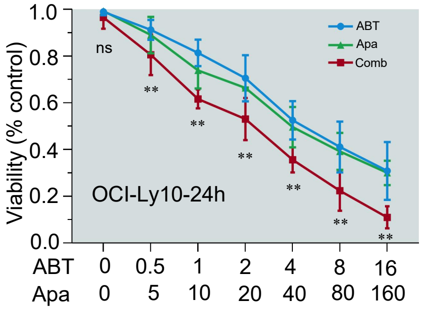


| ABT199 (nM) | Apatinib (μM) | FA | CI |
|---|---|---|---|
| 0.5 | 5 | 0.13 | 1.258 |
| 1 | 10 | 0.41 | 0.515 |
| 2 | 20 | 0.40 | 0.545 |
| 4 | 40 | 0.68 | 0.679 |
| 8 | 80 | 0.71 | 0.466 |
| 16 | 160 | 0.93 | 0.411 |

E

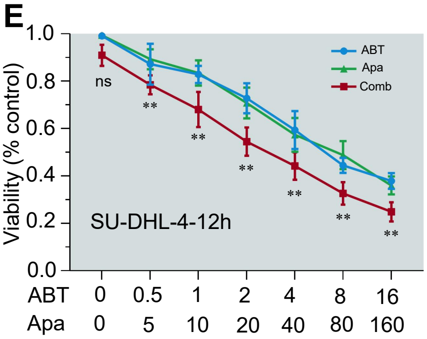


| ABT199 (nM) | Apatinib (μM) | FA | CI |
|---|---|---|---|
| 0.5 | 5 | 0.24 | 0.338 |
| 1 | 10 | 0.37 | 0.428 |
| 2 | 20 | 0.49 | 0.597 |
| 4 | 40 | 0.54 | 0.675 |
| 8 | 80 | 0.68 | 0.845 |
| 16 | 160 | 0.77 | 0.919 |

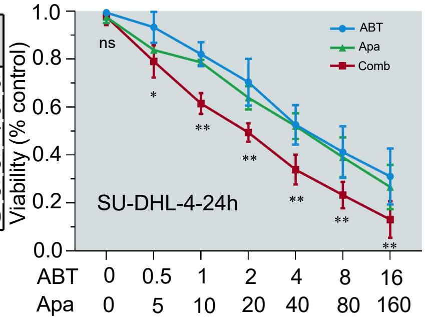


| ABT199 (nM) | Apatinib (μM) | FA | CI |
|---|---|---|---|
| 0.5 | 5 | 0.25 | 0.430 |
| 1 | 10 | 0.35 | 0.520 |
| 2 | 20 | 0.39 | 0.871 |
| 4 | 40 | 0.61 | 0.694 |
| 8 | 80 | 0.92 | 0.191 |
| 16 | 160 | 0.98 | 0.095 |
