# Supplementary figures and images for: Apatinib enhances chemosensitivity of ABT‐199 in diffuse large B‐cell lymphoma
Source: Mol Oncol. 2022 Sep 7;16(20):3735–53. doi: 10.1002/1878-0261.13309 (PMC9580892; doi:10.1002/1878-0261.13309)

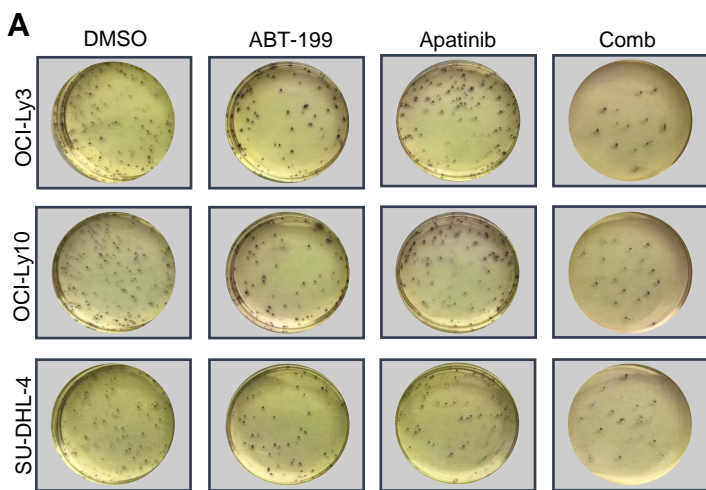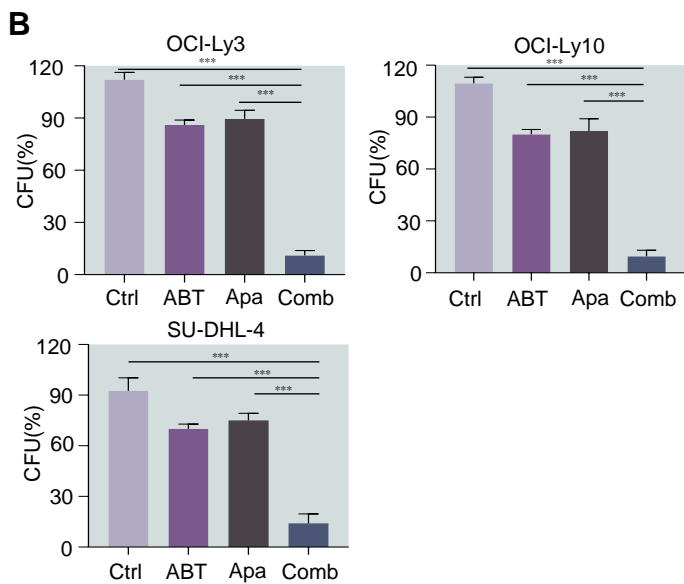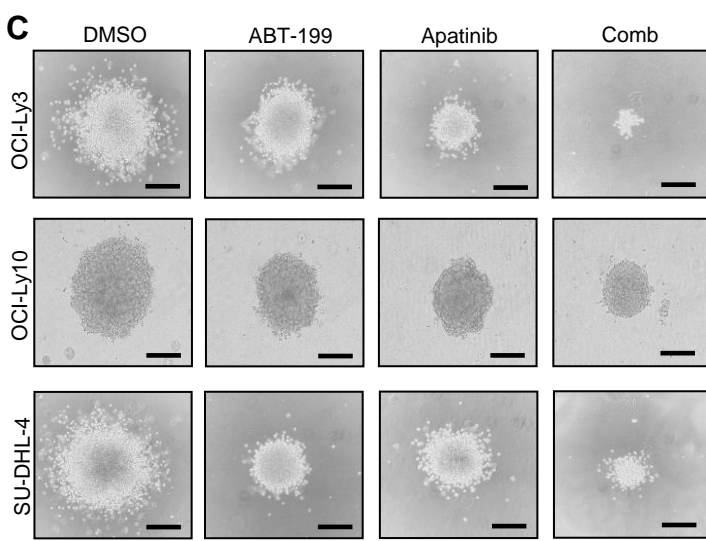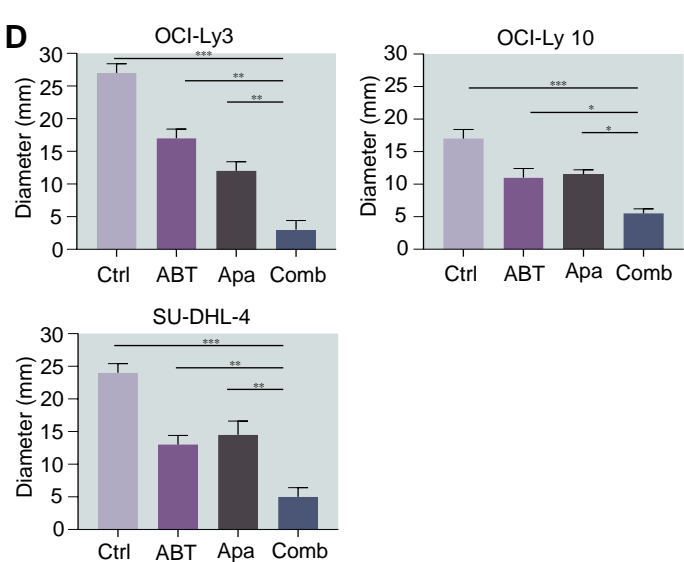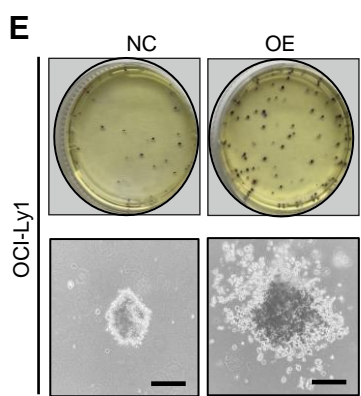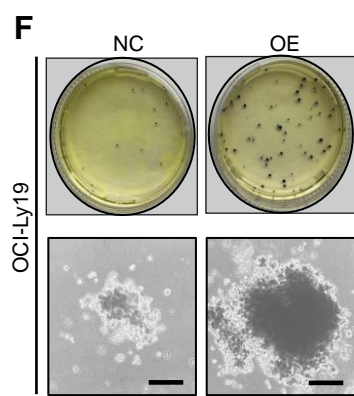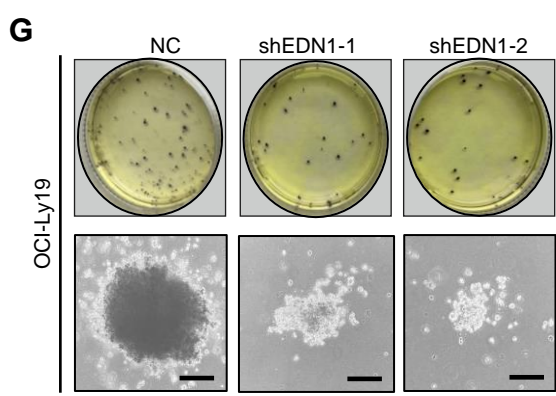

Supplement: Supplementary file 2 — Fig. S2. Co‐exposure to ABT‐199 and Apatinib inhibit cell proliferation in DLBCL cells. [file MOL2-16-3735-s006.pdf]

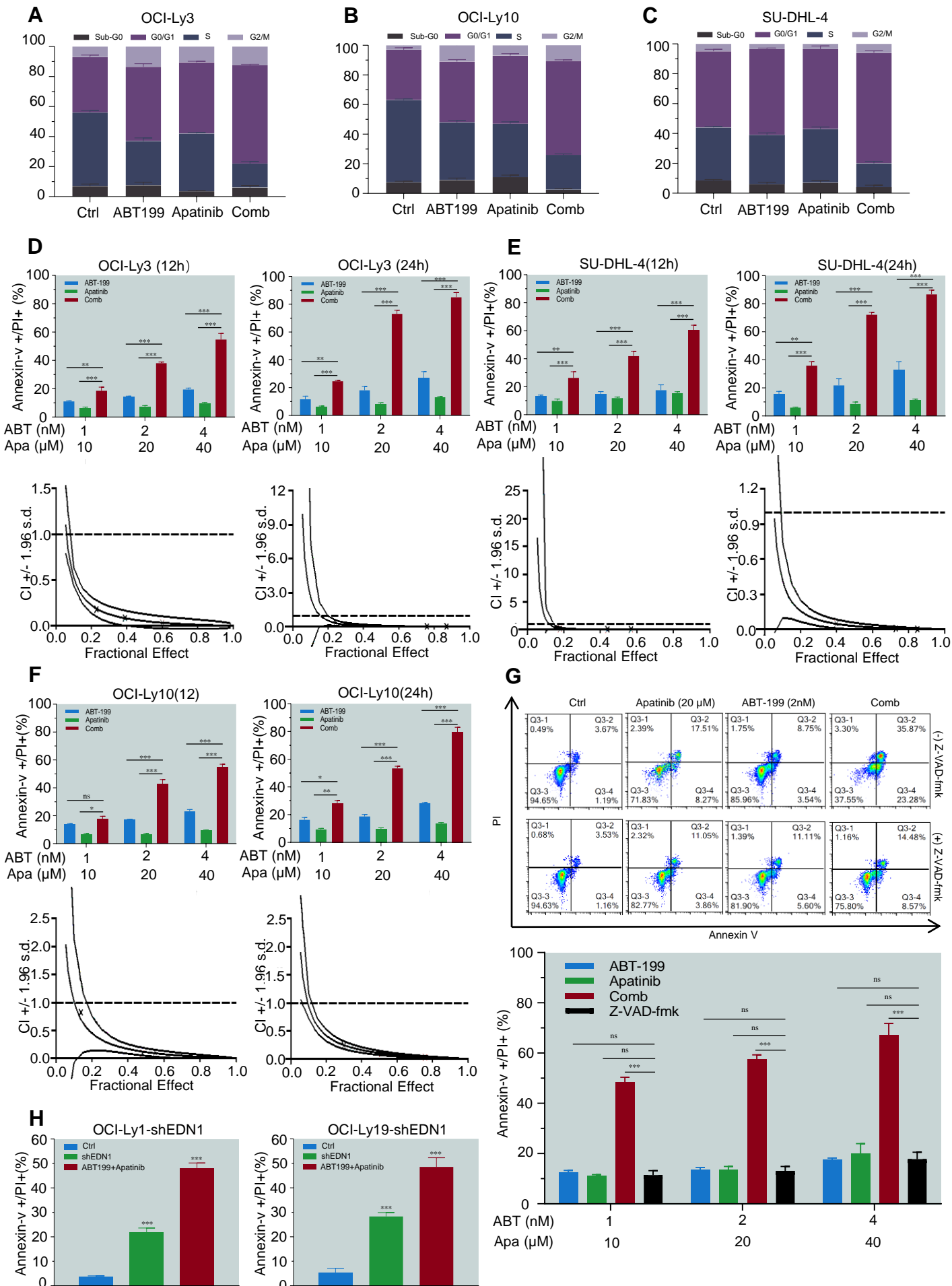

Supplement: Supplementary file 3 — Fig. S3. ABT‐199 combined with Apatinib induces apoptosis of DLBCL cells. [file MOL2-16-3735-s005.pdf]

A

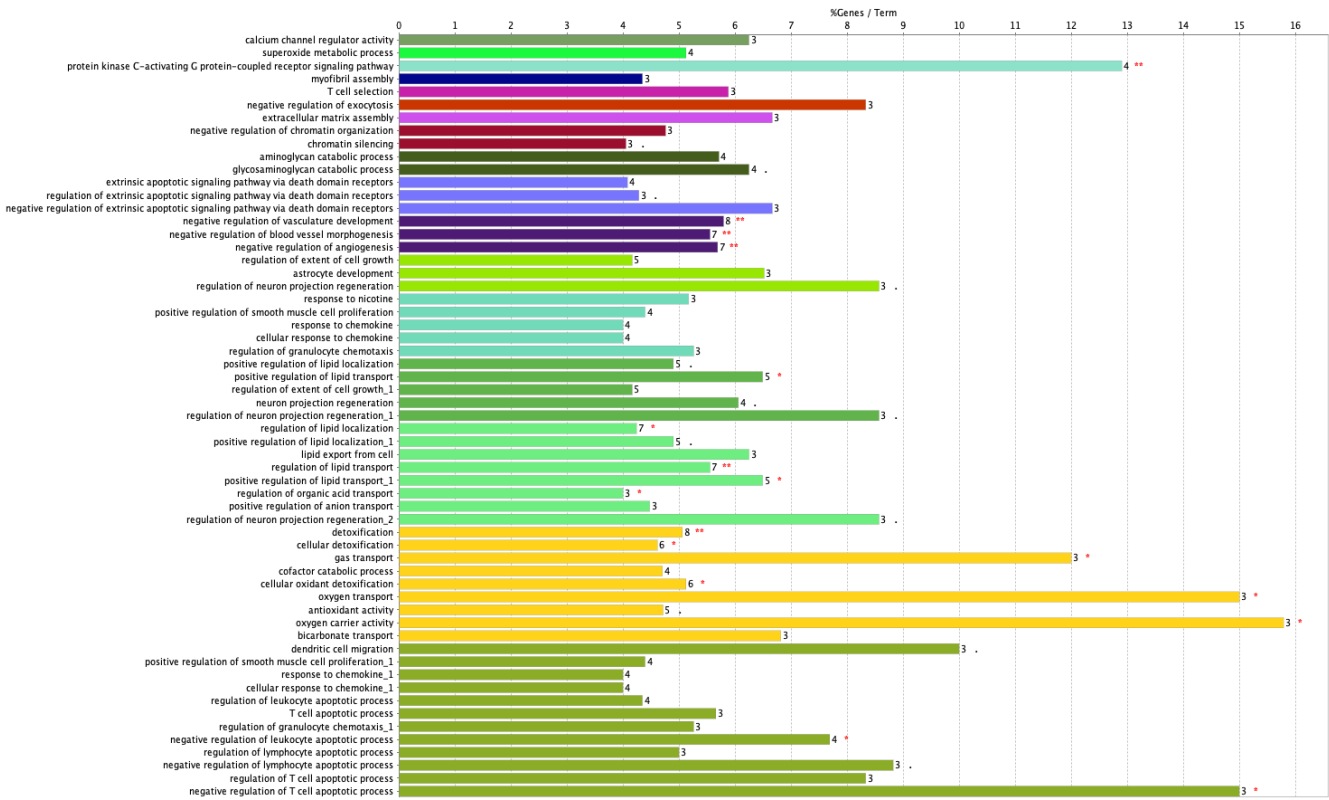

B

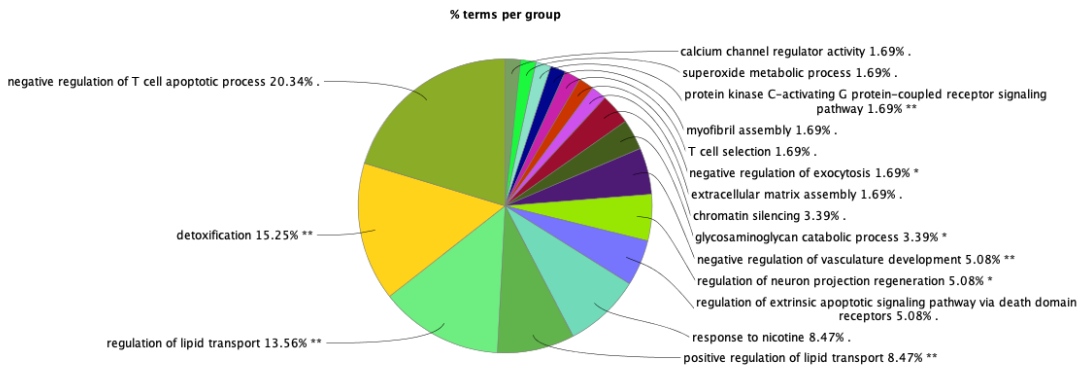

C

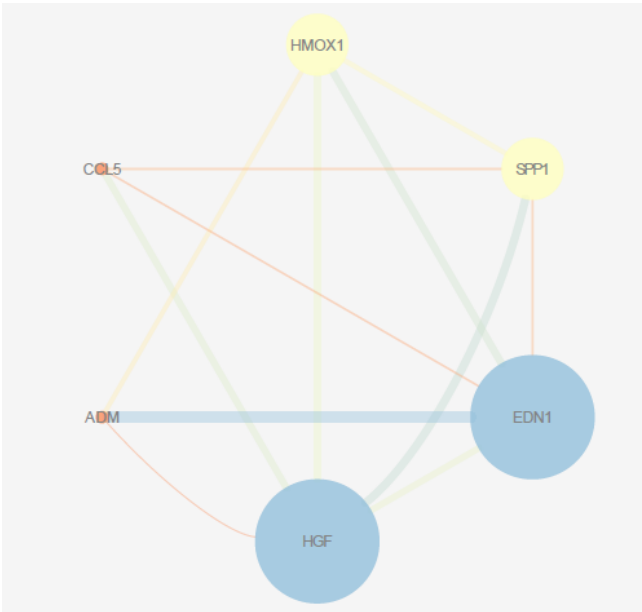

Supplement: Supplementary file 4 — Fig. S4. The percentage of GO terms in the ABT‐199 and/or Apatinib (all DEGs) treatment group is shown in the pie chart from the DLBCL cells transcriptome (all DEGs). [file MOL2-16-3735-s001.pdf]
