# Supplementary material for: Apatinib enhances chemosensitivity of ABT‐199 in diffuse large B‐cell lymphoma
Source: Mol Oncol. 2022 Sep 7;16(20):3735–53. doi: 10.1002/1878-0261.13309 (PMC9580892; doi:10.1002/1878-0261.13309)
Supplement: Supplementary file 6 — Legends [file MOL2-16-3735-s002.docx]

**Fig. S. 1** ABT-199 potentiates the anti-tumor activity of Apatinib toward various DLBCL cell lines. **(A)** ABT-199 resistance is associated with a variety of gene mutations. On this basis, we enriched the mutated genes by GO. **(B)** Changes in morphology in OCI-Ly3, OCI-Ly10, and SU-DHL-4 cells were incubated with ABT-199 and Apatinib alone or combination treatment for 24 h and visualized using an inverted microscope. Scale bar: 20 μm. **(C, D, E)** The inhibition rate of cell viability was measured at 12 h and 24 h in OCI-Ly3, OCI-Ly10, and SU-DHL-4 cell lines using the CCK-8. ns, no significant. Data are presented as mean ± SEM. Statistical analyses were performed using unpaired Student’s t tests. All experiments were repeated three times (n=3). *P < 0.05 and **P < 0.01.

**Fig. S. 2** ABT-199 combined with the Apatinib to inhibit cell clonogenicity. **(A, B)** OCI-Ly3, OCI-Ly10, and SU-DHL-4 cells were treated with ABT-199 (2 nM and Apatinib 20 μM for 24 h, after which the clonogenicity assay was performed to determine the percentage of CFU (left, representative images; right, bar graphs). Values indicate mean ± SD for at least three independent experiments performed in triplicate (*P < 0.05, **P < 0.01, and ***P < 0.001). **(C, D)** An inverted microscope observes the CFU size and measures its diameter (left, representative images; right, bar graphs). **(E, F)** When the EDN1 is overexpressed the clonogenicity assay was performed in OCI-Ly1 and OCI-Ly19 cells to determine the percentage of CFU. **(G)** After the EDN1 is knocked out the clonogenicity assay was performed in OCI-Ly19 cells to determine the percentage of CFU. Data are presented as mean ± SEM. Statistical analyses were performed using unpaired Student’s t tests. All experiments were repeated three times (n=3). *P < 0.05, **P < 0.01, and ***P < 0.001.

**Fig. S. 3** ABT-199 and Apatinib induce apoptosis of DLBCL cells, in association with altered cell cycle distribution. **(A, B, C)** Cell cycle distribution was assessed at 24 h by flow cytometry (black, dark purple, black-purple, and lavender present the statistical significance of Sub-G0, G0/G1, S, G2/M between DMSO and Combo group, respectively). **(D, E, F)** DLBCL cells were treated with ABT-199 and Apatinib alone or in combination for 12 and 24 h, after which the percentage of Annexin-V+ apoptotic cells were determined by flow cytometry after Annexin-V and PI double staining. The combination index (CI) was calculated based on apoptosis using the CalcuSyn software to evaluate the interaction between ABT-199 and Apatinib in DLBCL cell lines (CI < 1.0= 1.0, and >1.0, indicating synergistic, additive, and antagonistic effect, respectively). **(G)** Apoptosis of DLBCL cells were measured by Annexin-V and PI double staining after pretreatment with 20 μmol/L ZVAD-fmk for 2 h, followed by the indicated concentrations of ABT-199 and Apatinib for an additional 24 h. (H) The apoptosis rate of DLBCL cells after EDN1 knockdown and treatment with ABT-199 combined with Apatinib after EDN1 knockdown. Data are presented as mean ± SEM. Statistical analyses were performed using unpaired Student’s t tests. All experiments were repeated three times (n=3). *P < 0.05, **P < 0.01, and ***P < 0.001.

**Fig. S. 4** DLBCL cells transcriptome determined by RNA sequencing. **(A)** Gene ontology (GO) analysis for differentially expressed genes treated with ABT-199 or Apatinib for 24 h (all DEGs). **(B)** The percentage of GO terms in the combination treatment group is shown in the pie chart from the DLBCL cells transcriptome (all DEGs). **(C)** Five KEY genes in the combination treatment group have been dug using Cytoscape MCODE (P < 0.05).

**Table S1** 189 genes were differentially expressed after co-treatment ABT-199 and Apatinib compared with that in the single-treated samples.
